# A Proper Recognition

**Published:** 1881-04

**Authors:** 


### A PROPER RECOGNITION.

The degree of Doctor of Medicine was conferred by Rush Medical College, at its recent annual commencement, upon Dr. W. W. Allport, of Chicago. This is a worthily bestowed honor; one that might have been given long ago, at least so far as the merit of the recipient is concerned. Dr. Allport has stood in the front ranks of the dental profession for the last twenty years and has been a persistent advocate and worker for higher professional education and attainments in all respects. He has enjoyed the high esteem of the best men of the medical profession in his vicinity for many years, the culmination of which is shown in this recent act.

Long may he live to enjoy his honors.

